# Functional State of Rat Heart Mitochondria in Experimental Hyperthyroidism

**DOI:** 10.3390/ijms222111744

**Published:** 2021-10-29

**Authors:** Natalya Venediktova, Ilya Solomadin, Anna Nikiforova, Vlada Starinets, Galina Mironova

**Affiliations:** 1 Laboratory of Mitochondrial Transport, Institute of Theoretical and Experimental Biophysics Russia Academy of Sciences, 142290 Pushchino, Russia; nikiforanna@yandex.ru (A.N.); vlastar@list.ru (V.S.); mironova40@mail.ru (G.M.); 2 NPO PRIBOR GANK LLC, 123022 Moscow, Russia; iliusmaster@rambler.ru; 3 Department of Biochemistry, Cell Biology and Microbiology, Mari State University, 424001 Yoshkar-Ola, Russia

**Keywords:** mitochondria, energy metabolism, respiratory complexes, thyroid hormones, oxidative stress, mitochondrial dysfunction

## Abstract

In this work, the effect of thyroxine on energy and oxidative metabolism in the mitochondria of the rat heart was studied. Hyperthyroidism was observed in experimental animals after chronic administration of T_4_, which was accompanied by an increase in serum concentrations of free triiodothyronine (T_3_) and thyroxine (T_4_) by 1.8 and 3.4 times, respectively. The hyperthyroid rats (HR) had hypertrophy of the heart. In HR, there was a change in the oxygen consumption in the mitochondria of the heart, especially when using palmitoylcarnitine. The assay of respiratory chain enzymes revealed that the activities of complexes I, I + III, III, IV increased, whereas the activities of complexes II, II + III decreased in heart mitochondria of the experimental animals. It was shown that the level of respiratory complexes of the electron transport chain in hyperthyroid rats increased, except for complex V, the quantity of which was reduced. The development of oxidative stress in HR was observed: an increase in the hydrogen peroxide production rate, increase in lipid peroxidation and reduced glutathione. The activity of superoxide dismutase in the heart of HR was higher than in the control. At the same time, the activity of glutathione peroxidase decreased. The obtained data indicate that increased concentrations of thyroid hormones lead to changes in energy metabolism and the development of oxidative stress in the heart of rats, which in turn contributes to heart dysfunction.

## 1. Introduction

Currently, thyroid diseases are among the most abundant endocrine pathologies in the world [1]. Depending on the balance of thyroid hormones (TH) in the blood, the body’s metabolism can be either accelerated or slowed down on the contrary. TH play an important role in the metabolism, growth, and differentiation of tissues [2]. The mechanisms of action of these hormones are widely studied, but much remains to be learned about how TH regulate a variety of cellular functions. 

TH have a profound effect on the mitochondria, the organelles responsible for producing energy for the cell. Some studies have shown that an increased concentration of thyroid hormones can induce mitochondrial biogenesis, enhancing the ability of cells to generate the energy necessary for biological processes [3,4,5]. It is also known that there is a stimulation of the mitochondrial respiration rate of different organs [6,7,8] and a change in the activity of electron transport chain enzymes (ETC) [9]. With thyrotoxicosis, the generation of reactive oxygen species may increase [9,10,11], lipid oxidation and lipid composition disorders may occur [3,12,13], and changes in antioxidant defense in different tissues may be induced [10,11,14]. However, there is neither a clear understanding of the changes nor assessment of the inhibiting and activating the enzymes both of the antioxidant system and of the mitochondrial respiratory chain in different tissues with excessive levels of thyroid hormones.

Over the past decades, several new aspects have emerged in the possibility of studying mitochondria (biogenesis and mitophagy, the organization of the respiratory chain into supercomplexes), which provide new approaches for studying the complex relationship between the thyroid gland and the mitochondrial compartment [15,16,17]. For example, the effect of thyroxine on individual complexes and supercomplexes of the electron transport chain and their relationship with energy and oxidative metabolism of hyperthyroidism is still not fully studied. 

This work is a comprehensive study of the energy metabolism and the oxidative status of the mitochondria of the heart in rats with experimentally induced hyperthyroidism. The results obtained in this work will contribute to a deeper understanding of the processes underlying metabolic disorders in thyrotoxicosis, as well as contribute to the search for new targets for the correction of this pathology. The results obtained in this work will contribute to a deeper understanding of the processes underlying metabolic disorders in thyrotoxicosis, as well as contribute to the search for new targets for the correction of this pathology.

## 2. Results

### 2.1. Characteristics of Animals with Experimentally Induced Hyperthyroidism

The development of hyperthyroidism was confirmed by the determination of plasma T_3_ and T_4_ concentrations. There was about a 1.8- and 3.4-fold increase in T_3_ and T_4_ levels in HR compared with the values in the control group (Table 1). In addition, the administration of thyroxine to animals caused a decrease in body weight and heart (Table 1). The body weight gain in HR was reduced by more than two times. The heart/body weight ratio increase in HR is indicative of significant T_4_-induced cardiac hypertrophy (Table 1).

### 2.2. Determination of Mitochondrial DNA in Heart of Control and Hyperthyroid Rats

**Figure 1:**
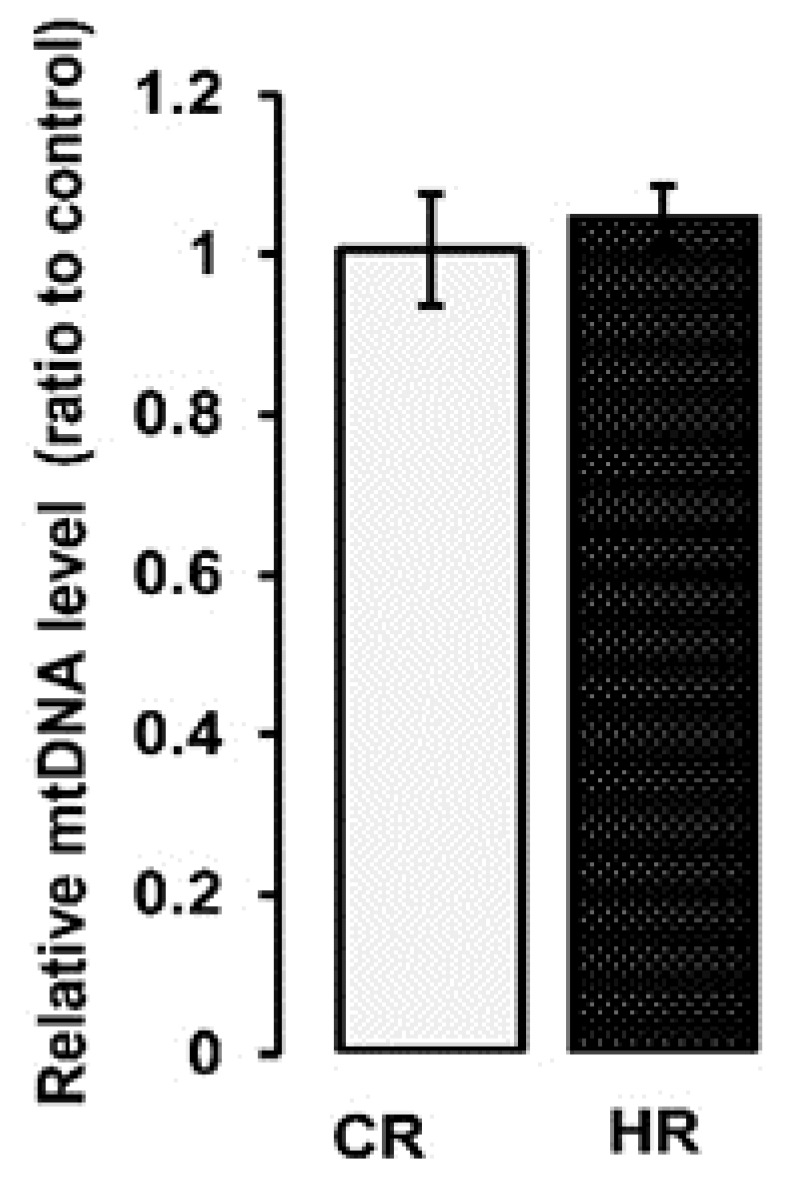
The relative mtDNA levels in the HM of animals. A real-time qPCR was carried out to determine the mtDNA copy number, which is calculated as the ratio of mitochondrial DNA (tRNA) to nuclear DNA (GAPDH) (*n* = 5 in each group). CR-control, HR-hyperthyroidism.

We determined the level of mitochondrial DNA in the heart tissue, indirectly reflecting the number of mitochondria. There were no changes in the increase in mtDNA content in the mitochondria of the heart of the control and hyperthyroid groups of animals (Figure 1). In addition, we measured the activity of citrate synthase (CS) (1386 ± 28 vs. 1353 ± 61 nmol/min_*_mg), which is usually used as a marker of the integrity/number of mitochondria [18]. The activity of this enzyme did not change in the heart mitochondria (HM) of both groups (Table 4). These data indicate that intraperitoneal administration of T_4_ (at a dose of 100 µg/100 g of body weight) did not lead to a change in the number of heart mitochondria in HR.

### 2.3. Respiratory Activity in Liver Mitochondria of Control and Hyperthyroid Rats

We estimated the functional state of rat heart mitochondria by measuring respiration rates in different metabolic states using NAD-/FAD-dependent substrates and TMPD (nonphysiological electron-donating compound)/ascorbate (Table 2 and Table 3). As can be seen from Table 2, changes in the mitochondrial respiration rate of HR occurred only in state_4_ (after all ADP was depleted). Accordingly, the respiratory control ratio (RCR) was reduced in heart mitochondria of hyperthyroid rats (HHM) by 15–20% (Table 2).

Table 3 shows that the development of hyperthyroidism led to a decrease in respiratory rates in different metabolic states from 7 to 28%, RCR by 29%, as well as to an increase in the time of phosphorylation of ADP by 26% when using L-palmitoylcarnitine as a substrate. A similar pattern was observed in the functioning of heart mitochondria of rats (RHM) using a substrate for complex IV, TMPD. The rates of substrate oxidation in state 3 and uncoupled respiration were reduced by 11 and 13% and RCR was decreased by 7%.

### 2.4. Mitochondrial Enzymatic Activities Linked to Energy Metabolism

The change in the parameters of the functioning of HHM could be associated with changes in the activity of electron-transport chain complexes. We measured the activity of individual complexes and complexes I + III, II + III in the mitochondria of the heart (Table 4). It was shown that the activity of complexes I (CI), III (CIII), IV (CIV) and complex I + III (CI + III) were increased from 24 to 32% compared to the control values. At the same time, the activities of complex II (CII) and complex II + III (CII + III) were decreased by 24% in hyperthyroid rats. The activity of CS and ATP-ase (CV) did not change (Table 4).

### 2.5. Level of Enzymes of the Electron Transport Chain in Heart Mitochondria of Control and Hyperthyroid Rats

**Figure 2:**
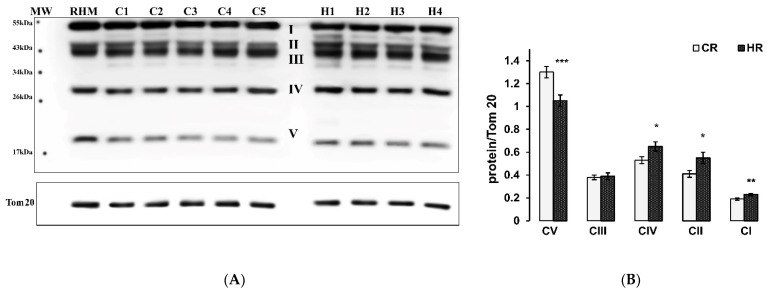
Level of representative subunits of the complexes in control and hyperthyroid rats. (**A**). Representative Western blot of OXPHOS subunits (I-ATP5A (CV), II-UQCRC2 (CIII), III-MTCO1 (CIV), IV-SDHB (CII) and V-NDUFB8 (CI)) was detected in isolated heart mitochondria (10 μg of protein/lane) by using the Total OXPHOS Rodent WB Antibody Cocktail. HRM-rat heart mitochondria western blot control, C1-C5-heart mitochondria from the control rats; H1-H4-heart mitochondria from the hyperthyroid rats. (**B**). Bar graphs represent the levels of appropriate complexes with respect to the Tom 20 level in absolute units. CR-control, HR-hyperthyroidism * *p* < 0.05, ** *p* < 0.02, *** *p* < 0.001 compared with the corresponding control data (*n* = 15 in each group).

In hyperthyroid animals, the levels of subunits of most of the studied ETC complexes were increased (Figure 2). So the quantity of the mitochondrial respiratory complexes IV, II, I was increased and the increases were as follows: complex I—22%, complex II—34%, complex IV—23% vs. the data in the control group. It was interesting to note a 24% decrease in the level of the CV subunit in HR (Figure 2).

### 2.6. Determination of Parameters Characteristic for Assessing the Development of Oxidative Stress

**Figure 3:**
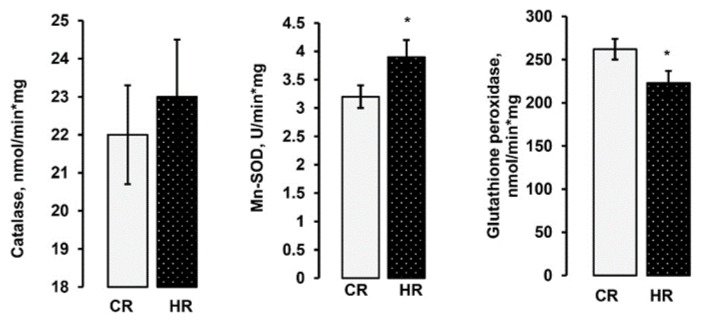
Activities of antioxidant enzymes in the heart mitochondria of control and hyperthyroid rats. CR-control, HR-hyperthyroidism. * *p* < 0.05, the difference is statistically significant (*n* = 15 in each group).

The mitochondrial respiration chain is an important site for reactive oxygen species (ROS) generation. Disorder in the balance between ROS formation and utilization leads to oxidative stress development. Therefore, we determined the activity of superoxide dismutase (SOD), catalase (CAT) and glutathione peroxidase (GP_X_), the rate of hydrogen peroxide formation, as well as the concentration of reduced glutathione (GSH_red_) and the degree of membrane lipid peroxidation the heart mitochondria of the studied rats. In the mitochondria of the HR, the activities of Mn-SOD (3.9 ± 0.3 vs. 3.2 ± 0.2 U/min·mg) were 22% higher than those in the control rats. The GPx activity in the heart mitochondria of HR (262 ± 12 nmol/min·mg) was decreased by 12% in comparison with the control values (233 ± 14 nmol/min·mg). In the mitochondria of the HR, the activity of CAT did not change (Figure 3).

Among the many antioxidants that exist in mitochondria, glutathione is involved in eliminating oxidative damage that leads to mitochondrial dysfunction and cell death. It was shown that glutathione metabolism was changed in animals after T_4_ treatment. There was a 63% and 66% decrease in the total and reduced GSH in HR as compared to the CR. The redox status (GSH/GSSG), an oxidative stress indicator, was reduced by 1.45 times in hyperthyroid rats. No significant differences were found between the groups of rats in the mitochondria of the heart with respect to the GSSG concentration (Table 5).

**Figure 4:**
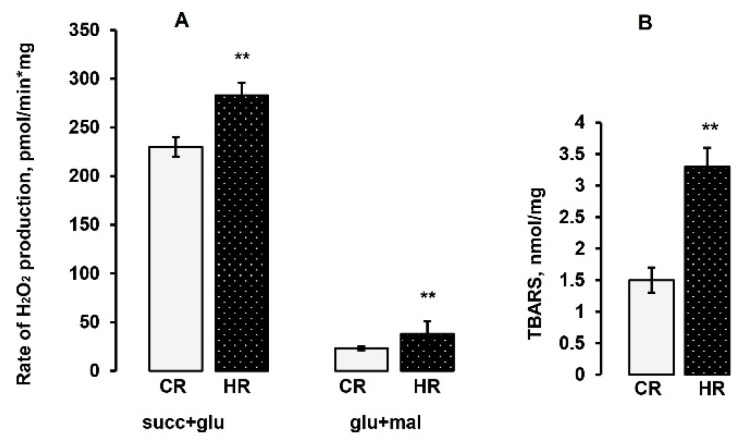
The rate of H_2_O_2_ production in heart mitochondria of control and hyperthyroid rats (**A**). The substrate used: 5 mM succinate/5 mM glutamate, 5 mM glutamate/5 mM malate. ** *p* < 0.02 compared with the control data (*n* = 10 in each group). (**B**). The TBARS concentration in heart mitochondria of control and hyperthyroid rats. ** *p* < 0.02 compared with the control data (*n* = 10 in each group). CR-control, HR-hyperthyroidism.

It was found that in the presence of succinate and glutamate, HM from control and hyperthyroid rats produced H_2_O_2_ with rates of 230 ± 10 and 283 ± 13 pmol/min_*_mg protein, respectively (23% increase in the hydrogen peroxide production rate in HHM). With the use of glutamate and malate as substrates, the H_2_O_2_ generation rate in HHM was significantly higher (60%) in comparison to the level observed in the heart mitochondria of control rats (CHM) (23.3 ± 2 vs. 38 ± 3.8 pmol/min_*_mg protein) (Figure 4A).

Heart mitochondria were assayed for oxidative damage. Lipid peroxidation was quantified by measuring the accumulation of thiobarbituric reacting substances. The TBARS assay quantifies the levels of malondialdehyde (MDA) and other minor aldehyde species through their reaction with thiobarbituric acid. As shown in Figure 4B, the TBARS concentration in HHM was more two times higher than in CHM (3.3 ± 0.3 vs. 1.5 ± 0.02 nmol/mg). Thus, the above data confirmed the development of oxidative stress in the heart mitochondria of the HR.

## 3. Discussion

Hyperthyroidism is a clinical syndrome caused by hyperfunction of the thyroid gland, associated with the production of a large number of thyroid hormones that have a toxic effect on the body. Excess TH in the blood causes an acute intensification of metabolism and leads to violations of the functioning of internal organs and systems [19]. Despite the long history of studying this pathology, many questions remain in its diagnosis and treatment. The basic and primary diagnostic tests in thyroid diseases include laboratory examination of T_3_ and T_4_. In our work, the thyroxine administration (100 µg/100 g body weight) caused an increase in the concentration of free T_3_ and T_4_ in the plasma of HR by several times, that indicated the development of hyperthyroidism in these animals. In addition, there was a decrease as body weight such and gain of weight in experimental animals, which is also a characteristic feature of hyperthyroidism. An increase in the ratio of heart mass to body weight revealed the development of cardiac hypertrophy in HR (Table 1). Hypertrophy can be a compensatory response to enhance contractility and preserve cardiac output. Nevertheless, persistent stress can drive this compensatory process into a pathological state, with reflective alterations in gene expression profile, contractile dysfunction, and extracellular remodeling [20].

Since thyroid hormones can enhance calorigenesis/thermogenesis by stimulating cellular respiration, mitochondria are a key link in the study of hyperthyroidism (HT). We have previously shown that in the liver mitochondria of HR there is an increase in respiration in different metabolic states with a slight decrease in the efficiency of oxidative phosphorylation and respiratory state ratio [21]. In the heart mitochondria of the HR, an increase in the rate of respiration was observed only in state_4_ during the work of CI and CII. In addition, the respiratory control ratio was reduced in HHM that could evidence some disorder of the coupling of oxidative phosphorylation or an increase in the rate of reverse electron transfer (Table 2). The heart is able to satisfy its energy needs through the oxidation of fatty acids, glucose, lactate and other oxidizable substrates. The use of palmitoylcarnitine/malate as a substrate showed that there was a decrease in the rate of phosphorylating (V_3_) and uncoupling (V_DNP_) respiration, as well as RCR in HHM. The rate of controlled respiration (V_4_) and the time of phosphorylation of ADP were increased (Table 3). At measuring the rate of oxygen consumption on CIV (TMPD/ascorbate), a decrease in respiration in state 3 and uncoupling (V_DNP_) respiration was detected, as well as a slight decrease in the RCR in HHM (Table 3). The assembly of respiratory complexes of the mitochondrial electron transport chain into respiratory supercomplexes provides kinetic benefits for the more efficient transport of electrons. The assumed structure of the respiratory supercomplex contributes to the fact that all subsequent components of the proximal CIV, which is in a fourfold excess with respect to CI, remain in an oxidized state regardless of the energy state of the mitochondria [22]. In this work, a decrease in respiratory rates on CIV was shown, which could affect a partial impairment of the coupling between ETC and oxidative phosphorylation in the mitochondria of the heart at experimentally induced hyperthyroidism. 

The change of respiration rates in HHM can be explained, for example, by a change in the level or activity of mitochondrial enzymes of the ETC in the animals. It was found that the activities of CI, CIII, CI + III, CIV were increased, whereas, CII and CII + III activities in the mitochondria of the heart HR were reduced. At the same time, the CS and CV activities did not change (Table 4). Despite the fact that the activity of most ETC complexes increased, there was no activation of respiration in the heart mitochondria of the HR (Table 2 and Table 3). The deficiency of ATP synthesis could be a consequence of either the failure of the proton pumps when they transfer electrons with a reduced extrusion of protons out across the membrane or switching respiratory chain complexes to the idle mode [4,23]. Because the electron transport in the mitochondrial ETC is a chain reaction, the disorder/decrease in the electron flow at any step can become a rate-limiting stage. In HR there was inhibition of only succinate dehydrogenase (CII) and succinate cytochrome c reductase (CII + III) activity (indirect evidence of coenzyme Q deficiency) in HM, which suggests impaired electron transfer from ubiquinone to cytochrome c and is likely to reverse electron transfer to CII and the possibility of reactive oxygen species formation. Probably, CII and CII + III are the most sensitive enzymes to the effect of high doses of TH in the mitochondria of the rat heart.

It was also shown that HR had an increase in the level of CI, CII, CIV subunits and a decrease in the CV subunit (Figure 2). The obtained data on the increase in the activity and quantity of most ETC complexes could be the result of either an increase in the number of mitochondria or an increase in the expression of genes responsible for the synthesis of these proteins. The mtDNA content did not change in the mitochondria of the heart of the control and hyperthyroid groups of animals (Figure 1). This was confirmed by the unchanged values of CS activity, the marker of the integrity/number of mitochondria (Table 4). In this case, thyroxine affected the expression of genes responsible for increasing the number of ETC proteins.

One of the suggestions about the mechanisms of the development of pathological processes at hyperthyroidism is considered to be oxidative stress in vivo [9,24,25]. The mitochondrial respiratory chain is the most important intracellular source of reactive oxygen species, but there is no unambiguous answer as to whether mitochondria-mediated oxidative stress in vivo is the main mechanism of HT. We consider that the observed alterations in the oxidative phosphorylation of HHM and changes in the activity of individual mitochondrial complexes led to the development of oxidative stress. The HT model caused an increase in the activity of Mn-SOD, which indirectly indicates a significant increase in the formation of the substrate of this enzyme—a superoxide radical (Figure 3). In addition, the rate of H_2_O_2_ formation in HHM was also increased in reactions with NAD-and FAD-dependent substrates (Figure 4A). At the same time, the activity of glutathione peroxidase in HHM was decreased (Figure 3). One of the main antioxidants that exist in the cell is glutathione. The importance of GSH is based not only on its abundance but also on its universality for detoxification of both hydrogen peroxide and preventing lipid peroxidation of cell membranes. The GSH concentration and the GSH/GSSG ratio decreased by approximately 70% in HHM (Table 5). We also found a significant increase in the degree of membrane peroxidation in HHM (Figure 4B). Thus, summarizing the above data, it can be stated that oxidative stress developed in the heart of rats with experimentally induced hyperthyroidism.

## 4. Materials and Methods

### 4.1. Animals

Wistar adult male rats weighing 210,230 g were used, aging 3–3.5 months. The animals were divided into control and hyperthyroid groups. All necessary procedures with laboratory rats were performed in specially equipped rooms of vivarium of ITEB RAS. Hyperthyroidism was induced by intraperitoneal injection of L-thyroxine at a dose of 100 μg per 100 g of animal weight for 7 days. The control rats (CR) were injected with an equal volume of saline. Rats were sacrificed by cervical dislocation at the same time. Plasma levels of free T3 and T4 were determined by an enzyme-linked assay (Vector-BEST, Russia). 

### 4.2. Quantification of Mitochondrial DNA

Total DNA (nuclear and mtDNA) was extracted from 10 mg of heart tissue using DNA-Extran 2 kit (Sintol, Russia) in accordance with the protocol of the manufacture. The concentration of total DNA was measured spectrophotometrically using a Nanodrop ND-1000 spectrophotometer (ND Technologies, Fremont, CA, USA). A total of 1 ng of the total DNA was taken for the reaction. The mtDNA level in the heart tissue was evaluated by PCR [26] and expressed as mtDNA/nuclear DNA ratio. For the assay, we selected the tRNA gene of the rat mitochondrial genome and the GAPDH gene of the nuclear one. Primers for mtDNA and nDNA are presented in Table 6. The Real-time PCR was performed with a QuantStudio 1 Real-Time PCR System (Thermo Fisher Scientific, UK) using the qPCRmix-HS SYBR reaction mixture (Eurogen, Russia), contained a commonly used fluorescent DNA binding dye SYBR Green II. A comparative CT method was used to quantify the results [27].

### 4.3. Isolation of Rat Heart Mitochondria (HM)

The heart was cleaned, cleared from blood vessels and perfused by ice-cold 0.9% NaCl. In order to isolate the total fraction of the HM (subsarcolemmal and interfibrillar), heart was perfused by the medium (0.225 M mannitol, 0.075 M sucrose, 0.01 M HEPES-KOH, 0.5 mM EGTA, 1.5 mg protease (P5380, Sigma)/1 g heart, pH 7.4). To neutralize the protease, the perfusion of the heart was repeated by the medium A (0.225 M mannitol, 0.075 M sucrose, 0.01 M HEPES-KOH, 0.2% fatty acid-free BSA, 0.5 mM EGTA, pH 7.4) and then by ice-cold 0.9% NaCl. After that, the heart was chopped and homogenized by Polytron disperser in the medium A (the ratio of tissue weight to isolation medium volume 1:10). The tissue homogenate was centrifuged at 700 × g for 5 min and the supernatant was sedimented at 12,000× *g* for 10 min. After the second centrifugation, the pellet was washed with the medium B (0.225 M mannitol, 0.075 M sucrose, and 0.01 M HEPES, pH 7.4) and centrifuged at 12,000× *g* for 10 min. The resulting pellet was resuspended in the medium B (the ratio of 0.1 ml of buffer per 1 g of the tissue). The concentration of the protein was determined by the Lowry method using bovine serum albumin as a standard [28]. 

### 4.4. Mitochondrial Respiration and Oxidative Phosphorylation

The rate of oxygen consumption was measured polarographically with a Clark-type gold electrode (Oxygraph-2k, OROBOROS Instruments, Innsbruck, Austria) at 26 °C under continuous stirring. The reaction medium contained 100 mM sucrose, 50 mM mannitol, 65 mM KCl, 10 mM HEPES, 0.5 mM EGTA-K, 2,5 mM KH_2_PO_4_, pH 7.4. The concentrations of substrates and other reagents were as follows: 5 mM potassium succinate, 2.5 or 5 mM potassium malate, 5 mM potassium glutamate, 0.02 mM L-palmitoylcarnitine, 0.5 mM TMPD, 2 mM ascorbate, 0.2 mM ADP, 0.05 mM 2,4-dinitrophenol (DNP) [21,22]. Energetic state definitions: V_4(0)_, state-basal substrate respiration; V_3_, state_3_-respiration stimulated by addition of ADP; V_4(1)_, state_4_-the metabolic state after all ADP is depleted; V_DNP_, state_U_-state of uncoupled respiration, respiration in the presence of the uncoupling agent DNP. The respiratory control ratio (RCR) was calculated as the ratio of respiration rates in state_3_/state_4_ [29]. The rates of oxygen consumption by mitochondria were expressed as nmol O_2_/min·mg. The concentration of mitochondrial protein in cuvette was 0.15–0.5 mg/mL.

### 4.5. Enzyme Activity Determination

Enzyme activities were determined in mitochondria as described earlier [21].

Enzyme activities were determined in mitochondria osmotically disrupted in 5 mM potassium phosphate buffer (pH 7.4) at 4 °C for 15 min and subjected to three freezing and thawing cycles. Then material was centrifuged at 14,000× *g* for 10 min. Activities of soluble enzymes were measured in the supernatant. The precipitate was lysed in 0.5 mL of a lysis mixture (5 mM potassium phosphate buffer, pH 7.4, protease inhibitor cocktail) and membrane-linked enzyme activity was determined in the lysate.

#### 4.5.1. SOD Activity

SOD activity was measured spectrophotometrically at 550 nm and 25 °C as the rate of inhibition of nitroblue tetrazolium reduction in the xanthine–xanthine oxidase system [30]. Activity unit (U) of SOD was taken as the enzyme quantity inhibiting reduction of nitroblue tetrazolium by 50%. Specific activity was defined as U/mg protein.

#### 4.5.2. CAT Activity

The catalase activity was estimated by measuring changes in absorbance at 240 nm and 25 °C using H_2_O_2_ as substrate and expressed as nmol/min·mg [31].

#### 4.5.3. GPX Activity

The glutathione peroxidase activity was estimated by measuring the decrease in absorbance at 340 nm due to the NADPH oxidation in the presence of H_2_O_2_ and GSH, expressed as nmol/min·mg protein [32].

#### 4.5.4. Respiratory Chain Enzyme Activities

The activities of respiratory chain complexes were determined in mitochondrial preparations according to methods [33] with modifications [21]. 

#### 4.5.5. Complex V (CV)

The assay relies on linking the ATPase activity to NADH oxidation via the conversion of phosphoenolpyruvate to pyruvate by pyruvate kinase (PK) and then pyruvate to lactate by lactate dehydrogenase (LDH). The mixture solution containing 0.3 mM NADH, 3 mM ATP, 2 mM PEP, 4 mM MgCl_2_, 4 mM KCl, 5 U LDH, 5 U PK, and 50 mM Tris–HCO_3_, pH 7.5. The reaction was started by adding mitochondrial extract. The change in absorbance was recorded over 3 min at 340 nm and 30 °C. Complex V activity is expressed as nmol/min_*_mg of protein.

### 4.6. Assay of the OXPHOS Complexes Level by Western Blot Analysis

To prepare samples for the determination of the level of OXPHOS Complexes, the aliquots (2 mg/mL) of native RHM were placed in an Eppendorf tube and solubilized in Laemmli buffer. The samples were heated to 37 °C for 3 min. Samples of equal protein concentrations (10 µg of mitochondrial protein) were applied to each lane and subjected to electrophoresis followed by Western blot analysis. Mitochondrial samples were separated by 12.5% SDS-PAGE and transferred to a nitrocellulose membrane. PageRuler Prestained Protein Ladder from Thermo Scientific (USA) was used as markers. 

Alterations in the levels of ETC enzymes were detected with Total OXPHOS Rodent WB Antibody Cocktail (ab 110413, monoclonal antibodies). The OXPHOS Antibody Cocktail consists of complex V ATP Synthase F1 Subunit Alpha (ATP5A-55 kDa), complex III Ubiquinol-Cytochrome C Reductase Core Protein 2 (UQCRC2-48 kDa), complex IV subunit Mitochondrially Encoded Cytochrome C Oxidase I (MTCO1-40 kDa), complex II subunit Succinate Dehydrogenase Complex Iron Sulfur Subunit B (SDHB-30 kDa), complex I subunit NADH:Ubiquinone Oxidoreductase Subunit B8 (NDUFB8-20 kDa). The Rat Heart Mitochondria Control (ab 110413, USA) was used as a Western blot control. 

The immunoreactivity was detected using the appropriate secondary antibody conjugated to horseradish peroxidase (Cell Signaling technology Inc., Danvers, MA, USA). The protein bands were quantified using a C-DiGit Blot Scanner (LI-COR Biotechnology, Lincoln, NE, USA) and Image Studio C-DiGit software. Tomm 20 protein content used for the normalization was not changed between the groups. Values protein bands are normalized to the Tomm 20 level in each sample.

### 4.7. Determination of the Rate of H_2_O_2_ Production

The rate of H_2_O_2_ production was detected using the system of the fluorescent dye Amplex red (excitation wavelength, 560 nm; emission wavelength, 590 nm) using a plate reader Tecan Spark 10 M (Tecan, Switzerland) at 37 °C under constant stirring. The incubation medium contained 100 mM sucrose, 50 mM mannitol, 65 mM KCl, 10 mM HEPES, 0.5 mM EGTA-K, 2.5 mM KH_2_PO_4_, pH 7.4 and a respiration substrate; then 2 U/ml of peroxidase, 10 μM AR, and 0.2 mg/mL of the mitochondrial protein were added to the reaction medium. The amount of the resulting hydrogen peroxide was calculated from the calibration curve. A standard hydrogen peroxide solution was prepared on the day of experiment; its concentration was determined using the molar absorption coefficient E240 = 43.6 M^−1^ cm^−1^.

### 4.8. Glutathione Assay

GSH was determined by the method based on oxidation of GSH by the sulfhydryl reagent 5,5′-dithio-bis(2-nitrobenzoic acid) (DTNB) to form the yellow derivative 5′-thio-2-nitrobenzoic acid (TNB), measurable at 412 nm. The glutathione disulfide (GSSG) formed can be recycled to GSH by glutathione reductase in the presence of NADPH [34]. The 25 µL of heart mitochondria was mixed with 0.1 mL of cold 10 mM HCl for the fixation of endogenous glutathione. The samples were centrifuged at 14.000× *g* for 5 min at 4 °C. Supernatant proteins (100 µL) were precipitated by 0.05 mL of cold 10% sulfosalicylic acid and removed by the second centrifugation. Supernatant was used for determination of the total GSH (GSH + GSSG). The incubation medium contained 100 mM Na_2_HPO_4_, pH 7.5, 5 mM EDTA, 0.2 mM NADPH, 0.6 mM DTNB, 0.6 U/ml of glutathione reductase. The reaction was initiated by adding the supernatant. Determination of oxidized glutathione (GSSG) concentration was performed in the supernatant after incubation with 2-vinylpyridine which interacts with GSH and prevents its oxidation to GSSG. The pH was checked with triethanolamine to 6–7 and after 60 min the samples were assayed as described above in the DTNB-GSSG reductase recycling method. Standard curves were prepared with GSH or GSSG and the contents were expressed as nmol/mg protein. The concentration of reduced glutathione was calculated as the difference between total GSH and GSSG. 

### 4.9. Estimation of Lipid Peroxidation

Lipid peroxidation was estimated by measuring levels of thiobarbituric acid reactive substances (TBARS) in heart mitochondria by the spectrophotometric method. The TBARS assay quantifies the levels of malondialdehyde (MDA) and other minor aldehyde species through their reaction with thiobarbituric acid. The TBARS concentration was calculated using the molar absorption coefficient of the colored complex TBA–MDA complex E_535_ = 1.56 × 105 M^−1^ cm^−1^ [35].

### 4.10. Statistical Analysis

The data were analyzed using the Graph Pad Prism 6 and Excel software and presented as means ± errors of the means (SEM) of 10–15 experiments (in control and experimental groups). Statistical differences between the data were determined by the two-tailed *t*-test.

## 5. Conclusions

The effect of thyroxine on the energy metabolism and the oxidative status of the heart mitochondria of the rat was studied. It was shown experimentally for the first time the activities and levels of enzyme subunits of the electron transport chain of heart mitochondria at HT: CI (NADH:ubiquinone oxidoreductase), CII (succinate dehydrogenase), CIII (ubiquinol cytochrome c oxidoreductase), CIV (cytochrome c oxidase) and CV (ATPase), as well as CI + III (NADH cytochrome c oxidoreductase) and CII + III (succinate cytochrome c reductase). Hyperthyroid rats had hypertrophy of the heart. A partial disturbance of the coupling between ETC and oxidative phosphorylation in the mitochondria of the heart and a decrease in the activity of CII/CII + III in experimentally induced hyperthyroidism in rats was shown, which in turn caused the development of oxidative stress. 

## Figures and Tables

**Table 1:** Thyroid hormone concentrations and morphometric parameters in control and hyperthyroid rats.

	Control	Hyperthyroidism	
T_3 free_, nmol/L	5.2 ± 0.1	9.3 ± 1.2 ***	78%↑
T_4 free_, nmol/L	19.2 ± 1.0	66.2 ± 4.4 ***	244%↑
Body weights, g	256 ± 3.3	236 ± 3.1 ***	8%↓
Body weight gain, g	36 ± 2.2	14 ± 2 ***	157%↓
Heart weights, g	0.9 ± 0.02	1.2 ± 0.03 ***	33%↑
Heart/body weight (×10^3^)	3.4 ± 0.06	4.8 ± 0.13 ***	41%↑

*** *p* ˂ 0.001 compared with the control data (*n* = 10 in each group). Changes induced by thyroxine: ↑—increase, ↓—decrease.

**Table 2:** Respiration parameters of heart mitochondria of control and hyperthyroid rats.

Succ + glu				Glu + mal		
	**CR**	**HR**		**CR**	**HR**	
V_4(0)_	44 ± 1.1	45 ± 1.5	-	10.7 ± 0.3	10.8 ± 0.3	-
V_3_	211 ± 6.5	201 ± 7	-	94 ± 2.4	94 ± 2.8	-
V_4(1)_	46 ± 1	53 ± 2 *	15%↑	12.5 ± 0.5	16 ± 0.5 **	24%↑
V_DNP_	210 ± 6.5	192 ± 8	-	97 ± 4.3	93.5 ± 2.6	-
RCR (V_3_/V_4_)	4.5 ± 0.1	3.9 ± 0.1 **	15%↓	7.5 ± 0.2	6.2 ± 0.2 ***	21%↓
t, s	67 ± 3.3	76 ± 3 *	13%↑	50.2 ± 1.3	57.1 ± 2.1 *	14%↑

V_4(0)_, V_3_, V_4(1)_, V_DNP_-rate of respiration in different metabolic state-nmol O_2_/min·mg of protein, t-sec. Additions: 5 mM succinate + 5 mM glutamate, 5 mM glutamate + 5 malate, 0.2 mM ADP, 0.05 mM DNP, Mitochondrial protein: 0.25 mg/mL in case of succ + glu or 0.5 mg/mL in case of glu + mal. CR-control, HR-hyperthyroidism. Changes induced by thyroxine: ↑—increase, ↓—decrease, -no change. * *p* < 0.05, ** *p* < 0.01, *** *p* < 0.001, the difference is statistically significant (*n* = 15–20 in each group).

**Table 3:** Respiration parameters of heart mitochondria of control and hyperthyroid rats.

Palm-carn + mal				TMPD + asc		
	**CR**	**HR**		**CR**	**HR**	
V_4(0)_	29 ± 1.5	31 ± 1	-	229 ± 4	212 ± 7	-
V_3_	265 ± 5	247 ± 5.2 *	7%↓	337 ± 5.5	303 ± 9.5 *	11%↓
V_4(1)_	32 ± 0.6	41 ± 1.4 ***	28%↑	217 ± 5	206 ± 6.5	-
V_DNP_	280 ± 7	261 ± 3.9 *	7%↓	341 ± 5.5	302 ± 8.5 **	13%↓
RCR (V_3_/V_4_)	8 ± 0.2	6.2 ± 0.2 ***	29%↓	1.6 ± 0.02	1.5 ± 0.03 *	7%↓
t, s	38.2 ± 1.1	48 ± 1.8 ***	26%↑	82 ± 2.8	98 ± 4.6	-

V_4(0)_, V_3_, V_4(1)_, V_DNP_-rate of respiration in different metabolic state-nmol O_2_/min·mg of protein, t-s. Additions: 0.02 mM L-palmitoylcarnitine + 2.5 mM malate, 0.5 mM TMPD + 2 mM ascorbate, 0.2 mM ADP, 0.05 mM DNP, Mitochondrial protein, 0.5 mg/mL in case of palm-carn + mal or 0.15 mg/mL in case of TMPD + asc. CR-control, HR-hyperthyroidism. Changes induced by thyroxine: ↑—increase, ↓—decrease, -no change. * *p* < 0.05, ** *p* < 0.01, *** *p* < 0.001, the difference is statistically significant (*n* = 15–20 in each group).

**Table 4:** Activities of enzymes of ETC and citrate synthase in heart mitochondria from control and hyperthyroid rats.

Enzymes	CR	HR	
CS, nmol/min·mg	1386 ± 28	1353 ± 61	-
CI, nmol/min·mg	376 ± 13	473 ± 17 *	26%↑
CII, nmol/min·mg	131 ± 3	100 ± 3 *	31%↓
CI + III, nmol/min·mg	563 ± 18	700 ± 25 **	24%↑
CII + III, nmol/min·mg	644 ± 30	488 ± 340 **	32%↓
CIII, nmol/min·mg	2481 ± 141	3170 ± 139 **	28%↑
CIV, nmol/min·mg	2616 ± 234	3459 ± 188 **	32%↑
CV, nmol/min·mg	3068 ± 105	2958 ± 116	-

* *p* < 0.05, ** *p* < 0.02, the difference is statistically significant (*n* = 15–20 in each group). CR—control, HR—hyperthyroidism. Changes induced by thyroxine: ↑: increase, ↓: decrease, -: no change.

**Table 5:** The concentrations of GSH_total_, GSH_red_, GSSG and GSH/GSSG ratio in heart mitochondria from control and hyperthyroid rats.

	CR	HR	
GSH_total_, nmol/mg	2.54 ± 0.3	1.56 ± 0.05 *	63%↓
GSH_red_, nmol/mg	2.47 ± 0.3	1.49 ± 0.05 *	66%↓
GSSG, nmol/mg	0.066 ± 0.01	0.065 ± 0.003	-
GSH/GSSG	40.8 ± 3.3	23.3 ± 1.7 **	75%↓

* *p* < 0.05, ** *p* < 0.02, the difference is statistically significant (*n* = 15 in each group). Changes induced by thyroxine: ↑—increase, ↓—decrease, -no change.

**Table 6:** List of gene-specific primers for the real-time PCR analysis.

Gene	Forward (5′ → 3′)	Reverse (5′ → 3′)
mt-tRNA	AATGGTTCGTTTGTTCAACGATT	AGAAACCGACCTGGATTGCTC
GAPDH	TGGCCTCCAAGGAGTAAGAAAC	GGCTCTCTCCTTGCTCTCAGTATC

## Data Availability

The data presented in this study are available on request from the corresponding author.

## Abbreviations

TH	Thyroid hormones
T_3_	triiodothyronine
T_4_	thyroxin
ETC	electron transport chain
ROS	reactive oxygen species
SOD	superoxide dismutase
GPx	glutathione peroxidase
CAT	catalase
CI	complex I
CII	complex II
CIII	complex III
CIV	complex IV
C I + III	complex I + III
C II + III	complex II + III
CS	citrate synthase
TBARS	thiobarbituric acid reactive substances
HHM	hyperthyroid heart mitochondria
CHM	control heart mitochondria
GSH	glutathione
GSSG	glutathione disulfide
TMPD	nonphysiological electron—donating compound

## References

[1] Heuck C.C., Kallner A., Kanagasabapathy A.S., Riesen W. (2000). Diagnosis and Monitoring of Diseases of the Thyroid.

[2] Cicatiello A.G., Di Girolamo D., Dentice M. (2018). Metabolic Effects of the Intracellular Regulation of Thyroid Hormone: Old Players, New Concepts. Front. Endocrinol..

[3] Lanni A., Moreno M., Goglia F. (2011). Mitochondrial Actions of Thyroid Hormone. Compr. Physiol..

[4] Pietrobon D., Azzone G.F., Walz D. (1981). Effect of Funiculosin and Antimycin A on the Redox-Driven H+-Pumps in Mitochondria: On the Nature of ‘Leaks’. JBIC J. Biol. Inorg. Chem..

[5] Lombardi A., Moreno M., De Lange P., Iossa S., Busiello R.A., Goglia F. (2015). Regulation of skeletal muscle mitochondrial activity by thyroid hormones: Focus on the “old” triiodothyronine and the “emerging” 3,5-diiodothyronine. Front. Physiol..

[6] Harper M.-E., Seifert E.L. (2008). Metabolic Effects of Thyroid Hormones Thyroid Hormone Effects on Mitochondrial Energetics. Thyroid.

[7] Lanni A., Moreno M., Lombardi A., De Lange P., Goglia F. (2001). Control of energy metabolism by iodothyronines. J. Endocrinol. Investig..

[8] Horrum M.A., Tobin R.B., Ecklund R.E. (1990). Thyroid hormone effects on the proton permeability of rat liver mitochondria. Mol. Cell. Endocrinol..

[9] Venditti P., Pamplona R., Portero-Otin M., De Rosa R., Di Meo S. (2006). Effect of experimental and cold exposure induced hyperthyroidism on H_2_O_2_ production and susceptibility to oxidative stress of rat liver mitochondria. Arch. Biochem. Biophys..

[10] Araujo A., Ribeiro M., Enzveiler A., Schenkel P., Fernandes T., Partata W., Irigoyen M., Llesuy S., Belló-Klein A. (2006). Myocardial antioxidant enzyme activities and concentration and glutathione metabolism in experimental hyperthyroidism. Mol. Cell. Endocrinol..

[11] Das K., Chainy G.B.N. (2004). Thyroid hormone influences antioxidant defense system in adult rat brain. Neurochem. Res..

[12] Moreno M., de Lange P., Lombardi A., Silvestri E., Lanni A., Goglia F. (2008). Metabolic Effects of Thyroid Hormone Derivatives. Thyroid.

[13] Shinohara R., Mano T., Nagasaka A., Hayashi R., Uchimura K., Nakano I., Watanabe F., Tsugawa T., Makino M., Kakizawa H. (2000). Lipid peroxidation levels in rat cardiac muscle are affected by age and thyroid status. J. Endocrinol..

[14] Huh K., Kwon T.-H., Kim J.-S., Park J.M. (1998). Role of the hepatic xanthine oxidase in thyroid dysfunction: Effect of thyroid hormones in oxidative stress in rat liver. Arch. Pharmacal Res..

[15] Goldenthal M.J., Weiss H.R., Marín-García J. (2004). Bioenergetic remodeling of heart mitochondria by thyroid hormone. Mol. Cell. Biochem..

[16] Letts J., Sazanov L. (2017). Clarifying the supercomplex: The higher-order organization of the mitochondrial electron transport chain. Nat. Struct. Mol. Biol..

[17] Gao F., Zhang J. (2018). Mitochondrial quality control and neurodegenerative diseases. Neuronal Signal..

[18] Reisch A.S., Elpeleg O. (2007). Biochemical Assays for Mitochondrial Activity: Assays of TCA Cycle Enzymes and PDHc. Methods Cell Biol..

[19] Moini J., Pereira K., Samsam M. (2020). Epidemiology of Thyroid Disorders.

[20] Elnakish M.T., Ahmed A.A.E., Mohler P.J., Janssen P.M.L. (2015). Role of Oxidative Stress in Thyroid Hormone-Induced Cardiomyocyte Hypertrophy and Associated Cardiac Dysfunction: An Undisclosed Story. Oxidative Med. Cell. Longev..

[21] Venediktova N.I., Mashchenko O.V., Talanov E.Y., Belosludtseva N.V., Mironova G.D. (2020). Energy metabolism and oxidative status of rat liver mitochondria in conditions of experimentally induced hyperthyroidism. Mitochondrion.

[22] Panov A. (2014). Practical mitochondriology. Pitfalls and Problems in Studies of Mitochondria with a Description of Mitochondrial Functions.

[23] Semenova A.A., Samartsev V.N., Dubinin M.V. (2021). The stimulation of succinate-fueled respiration of rat liver mitochondria in state 4 by α,ω-hexadecanedioic acid without induction of proton conductivity of the inner membrane. Intrinsic uncoupling of the bc complex. Biochimie.

[24] Asayama K., Dobashi K., Hayashibe H., Megata Y., Kato K. (1987). Lipid Peroxidation and Free Radical Scavengers in Thyroid Dysfunction in the Rat: A Possible Mechanism of Injury to Heart and Skeletal Muscle in Hyperthyroidism. Endocrinology.

[25] Venditti P., Di Stefano L., Di Meo S. (2010). Oxidative stress in cold-induced hyperthyroid state. J. Exp. Biol..

[26] Quiros P.M., Goyal A., Jha P., Auwerx J. (2017). Analysis of mtDNA/nDNA Ratio in Mice. Curr. Protoc. Mouse Biol..

[27] Schmittgen T.D., Livak K.J. (2008). Analyzing real-time PCR data by the comparative CT method. Nat. Protoc..

[28] Lowry O.H., Rosebrough N.J., Farr A.L., Randall R.J. (1951). Protein measurement with the folin phenol reagent. J. Biol. Chem..

[29] Chance B., Williams G. (1955). Respiratory enzymes in oxidative phosphorylation. J. Biol. Chem..

[30] Beauchamp C., Fridovich I. (1971). Superoxide dismutase: Improved assays and an assay applicable to acrylamide gels. Anal. Biochem..

[31] Aebi H.E., Bergmeyer H.U. (1984). Catalase. Methods of Enzymatic Analysis.

[32] Kosenko E., Kaminsky Y., Kaminsky A., Valencia M., Lee L., Hermenegildo C., Felipo V. (1997). Superoxide production and an-tioxidant enzymes in ammonia intoxication in rats. Free Radic. Res..

[33] Spinazzi M., Casarin A., Pertegato V., Ermani M., Salviati L., Angelini C. (2011). Optimization of respiratory chain enzymatic assays in muscle for the diagnosis of mitochondrial disorders. Mitochondrion.

[34] Rahman I., Kode A., Biswas S.K. (2006). Assay for quantitative determination of glutathione and glutathione disulfide levels using enzymatic recycling method. Nat. Protoc..

[35] Ohkawa H., Ohishi N., Yagi K. (1979). Assay for lipid peroxides in animal tissues by thiobarbituric acid reaction. Anal. Biochem..

